# The calendar of epidemics: Seasonal cycles of infectious diseases

**DOI:** 10.1371/journal.ppat.1007327

**Published:** 2018-11-08

**Authors:** Micaela Elvira Martinez

**Affiliations:** Climate & Health, Department of Environmental Health Sciences, Mailman School of Public Health, Columbia University, New York, New York, United States of America; Nanyang Technological University, SINGAPORE

## Introduction

**Fig 1:**
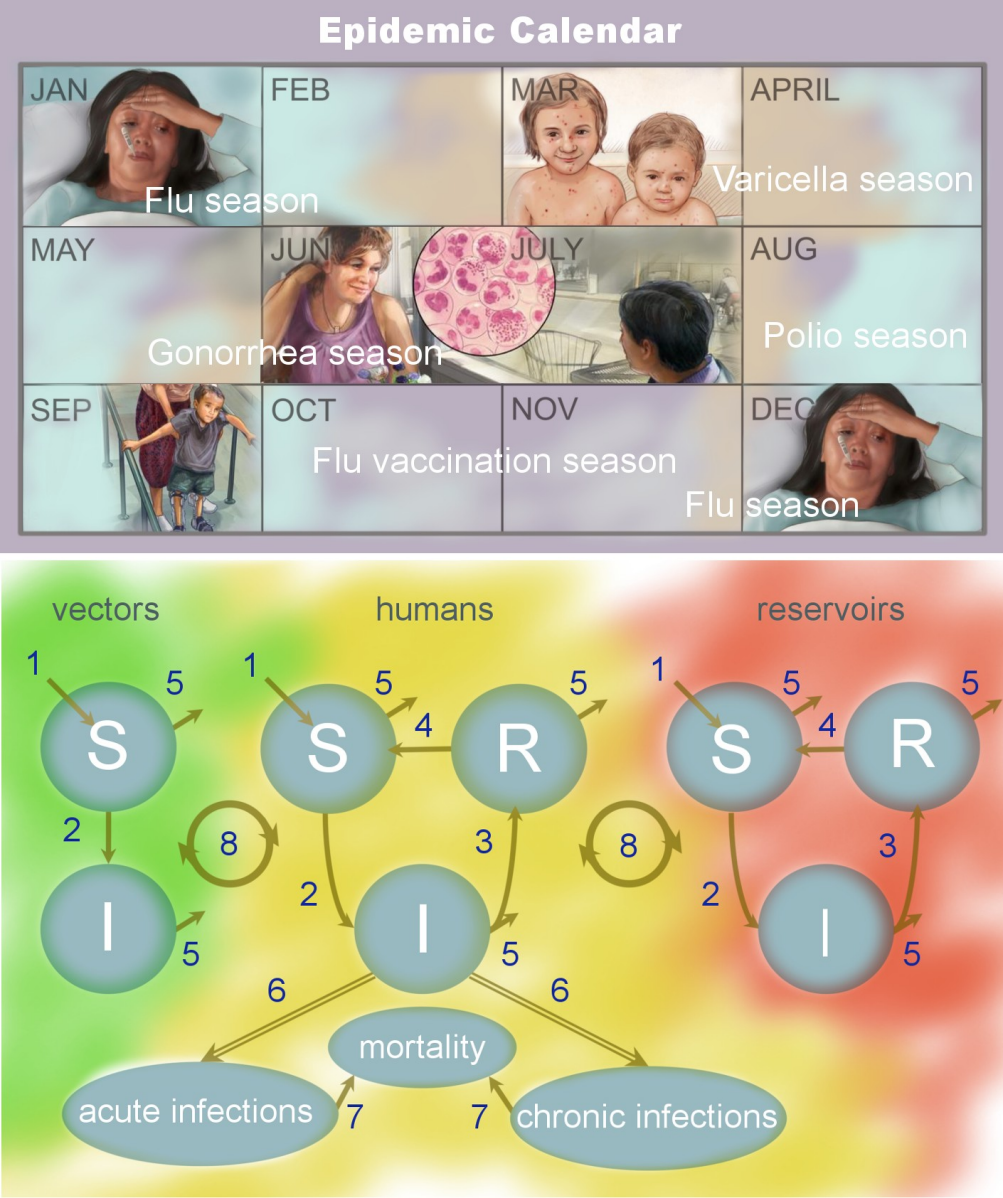
Epidemic calendar. The concept of an epidemic calendar is illustrated in the top panel. Infectious diseases are seasonal, especially the occurrence of acute and epidemic-prone diseases. In any given population, infectious diseases are distributed throughout the year. Annual cycles of infectious disease are a ubiquitous feature of infection (Tables 1–4). The illustration depicts the wintertime seasonality of flu, springtime peaks of varicella (i.e., chickenpox), and the summertime occurrence of gonorrhea and polio, in the Northern Hemisphere. The bottom panel is a SIR schematic for the transmission of human infectious diseases, which include acute and chronic diseases, those that are vector-borne, and those that are zoonotic with animal reservoirs. The vector, human, and reservoir hosts populations are partitioned into individuals who are susceptible to infection, infected, and recovered and immune. Seasonality may enter into any of the eight key elements of the system: (1) susceptible recruitment via reproduction, (2) transmission, (3) acquired immunity and recovery, (4) waning immunity, (5) natural mortality, (6) symptomatology and pathology (which may be acute or chronic, depending on the disease), (7) disease-induced mortality, and (8) cross-species transmission. *Disease illustrations reproduced from Google Medical Information*. I, infected; R, recovered and immune; S, susceptible.

Seasonal cyclicity is a ubiquitous feature of acute infectious diseases [1] and may be a ubiquitous feature of human infectious diseases in general, as illustrated in Tables 1–4. Each acute infectious disease has its own seasonal window of occurrence, which, importantly, may vary among geographic locations and differ from other diseases within the same location. Here we explore the concept of an epidemic calendar, which is the idea that seasonality is a unifying feature of epidemic-prone diseases and, in the absence of control measures, the local calendar can be marked by epidemics (Fig 1). A well-known example of a calendar marked by epidemics is that of the Northern Hemisphere, where influenza outbreaks occur each winter [2, 3] (hence the colloquial reference to winter as "the flu season"). In contrast, chickenpox outbreaks peak each spring [4, 5], and polio transmission historically occurred each summer [6].

**Table 1:** Seasonal drivers of human infectious diseases. Drivers categorized as being related to (a) vector seasonality, (b) seasonality in nonhuman animal host (i.e., livestock, other domestic animals, or wildlife), (c) seasonal climate (e.g., temperature, precipitation, etc.), (d) seasonal nonclimatic abiotic environment (e.g., water salinity), (e) seasonal co-infection, (f) seasonal exposure and/or behavior and/or contact rate, (g) seasonal biotic environment (e.g., algal density in waterbodies).

Infection/disease	Type	Seasonal driver(s)	Description
African sleeping sickness	Chronic	a	Tsetse fly distribution changes seasonally; expanded range during rainy season [7]
Anthrax	Acute	b	Zoonotic disease with seasonality reported in wildlife and livestock; seasonality varies among location and species [8]
Avian influenza	Acute	b	Winter in both humans and poultry (in Asia) [9, 10]
Bacterial Pneumonia	Acute	c, d, and e	Peaks in midwinter (in the US); it is associated with influenza [11]
Brucellosis	Acute	b	Spring and summer in wildlife and livestock; the timing relates to the birthing season; peaks in the summer in humans [12]
Buruli ulcer	Chronic	c	Varies by location; some studies have not observed seasonality [13]
Chagas disease	Acute and chronic	a	Peaks in spring and summer in countries with distinct seasons [14]
Chickenpox	acute	f	Peak in spring in the Northern and Southern Hemisphere [15]
Chikungunya	Acute	a	Rainy season when vector density peaks. [16]
Cholera	Acute	c, d, and g	Seasonality is stronger in countries further from the equator; outbreaks generally occur in warm months [17]
Crimean-Congo hemorrhagic fever	Acute	a	Seropositivity in livestock correlates with seasonal changes in tick parasitism; human cases correlate with livestock seropositivity [18]
Cryptosporidium	Acute	c	Increased risk of cryptosporidium associated with high ambient temperature and high rainfall [19]
Cutaneous leishmaniasis	Acute and chronic	a and b	Strong seasonal variation with elevated incidence from October to March (in Tunisia). Seasonality may be due to climate effects on the vector: blood-feeding sand flies [20]
Dengue fever	Acute	a	Rainy season (in Thailand) [21]
Diphtheria	Acute	f	Spring and summer (in Portugal) [22]
Dracunculiasis	Chronic	c, d, f, and g	Dry season (in Nigeria) [23]
Ebola	Acute	b	In wildlife the peak is in the dry season (in Gabon) [24]
Echinococcosis	Chronic	b	Exposure to livestock carrying the infection is seasonal [25]
*Escherichia coli* (pathogenic)	Acute	b	Seasonal in cattle; cattle are a source for human infection [26]
Foodborne trematodiases	Chronic	f	Exposure is seasonal due to seasonal ingestion of infected snails [27]
Genital herpes	Chronic	f	Elevated incidence in spring/summer and lower in winter (in the US) [28]
Gonorrhea	Chronic	f	Peak cases in the summer and autumn (in the US) [28]

Since seasonal timing may differ among geographic areas, study location is indicated in parentheses.

**Table 2:** Seasonal drivers of human infectious diseases (continued from Table 1). Drivers categorized as being related to (a) vector seasonality, (b) seasonality in nonhuman animal host (i.e., livestock, other domestic animals, or wildlife), (c) seasonal climate (e.g., temperature, precipitation, etc.), (f) seasonal exposure and/or behavior and/or contact rate, (g) seasonal biotic environment (e.g., algal density in waterbodies), (h) seasonal flare-up/symptoms and/or remission/latency, (i) observed seasonal incidence with no hypotheses regarding drivers.

Infection/disease	Type	Seasonal driver(s)	Description
Haemophilus influenzae	Acute	i	Slightly elevated incidence in winter (in the US) [29]
Hepatitis A	Acute	f and i	Dry season (in Brazil) [30, 31]
Hepatitis B	Chronic	h	Seasonality is observed with elevated levels in spring and summer and/or autumn in some parts of the world, whereas there is lack of seasonality in other parts of the world [31, 32]
Hepatitis C	Acute and chronic	f	Seasonality observed in some countries and absent in others; spring and/or summer peaks in Egypt, China, and Mexico while there is a winter peak in India [31]
Hepatitis E	Acute	c	Waterborne outbreaks occur during the rainy season or following flooding (in China) [33]
Herpes zoster (shingles)	Acute and chronic	i and h*	Highest in August and lowest in winter (in Japan) [34]
HIV	Chronic	g	There is some evidence to suggest there is seasonal variation in the progression to AIDS; hypothesized to be related to seasonal nutritional deficiencies (study done in Uganda) [35]
Influenza	Acute	c	Winter (in the Northern Hemisphere) [36]
Japanese encephalitis	Acute	a	It is seasonal in the northern part of the tropical zone; outbreaks happen at the end of the rainy season, but there is no seasonal pattern in tropical regions [37]
Lassa fever	Acute	c	Increase in the number of Lassa fever cases during the dry season (in Nigeria) [38]
Legionellosis	Acute	c	Peaks during hot months and particularly during humid periods (in the US) [39]
Leishmania	Chronic	a	Transmitted by sand flies; domestic dogs are the main reservoir, and they are exposed during a discrete transmission season [40]
Leprosy	Chronic	b	Armadillos are the reservoir, and antibody prevalence is seasonal within them [41]
Leptospirosis	Acute	c	Peaks when there is hot weather; usually in a rainy period (on all continents) [42]
Lyme disease	Acute and Chronic	a	Peaks in summer around the time of maximal activity of the nymphal stage of the tick vector (in the US) [43]
Lymphatic filariasis	Chronic	a and c	Transmission is intensified during the rainy season [44]
Malaria	Acute	a	There is a spectrum of seasonal strength; some regions have strong seasonality and no seasonality in others [45]

Since seasonal timing may differ among geographic areas, study location is indicated in parentheses.

*Indicated by author.

**Table 3:** Seasonality of human infectious diseases (continued from Tables 1 and 2). Drivers categorized as being related to (a) vector seasonality, (b) seasonality in nonhuman animal host (i.e., livestock, other domestic animals, or wildlife), (c) seasonal climate (e.g., temperature, precipitation, etc.), (f) seasonal exposure and/or behavior and/or contact rate, (g) seasonal biotic environment (e.g., algal density in waterbodies), (h) seasonal flare-up/symptoms and/or remission/latency, (i) observed seasonal incidence with no hypotheses regarding drivers.

Infection/disease	Type	Seasonal driver(s)	Description
Marburg	Acute	b	Seasonal incidence in bat reservoirs (in Uganda); seasonal peaks coincided with the twice-annual birthing season [46]
Measles	Acute	f	Elevated transmission driven by aggregation of children in school; seasonality in developing countries related to agricultural cycles [47, 48]
Meningococcal disease	Acute	c and h	Incidence varies seasonally in both tropical and temperate countries. Elevated incidence during the dry season (in sub-Saharan Africa). [49]
MERS-CoV	Acute	b	Introductions into humans are seasonal and are more frequent during the camel calving season. [50]
Onchocerciasis (river blindness)	Acute and chronic	a	Higher transmission potential in the rainy season when vector abundance and infection is elevated (in Nigeria) [51]
Pertussis	Acute	i and f*	Higher incidence June through October (in the US) [52]
Plague	Acute	a, b, c, f, and g	The seasonality varies among countries and is dependent on seasonality of reservoir and vector species and in some cases agricultural cycles [53]
Poliomyelitis	Acute	i, c*, and h*	Epidemics occurred during the summer (in the US) [6]
Rabies	Acute	b	Rabies is seasonal in bats, which are a source of human exposure [54]
RSV	Acute	i and c*	Peaks in winter months in temperate regions; less pronounced seasonality in the tropics [55]
Rift Valley fever	Acute	a and c	Associated with periods of heavy rainfall [56]
Rotavirus	Acute	i and c*	Geographical gradient in seasonality; peaks in December/January in the Southwest US and April/May in the Northeast US [57]
Rubella	Acute	f	Two seasonal peaks in transmission per year in Kenya; late-winter to early-summer peaks in the US [58, 59]
Salmonellosis	Acute	i	Increased number of isolates in the warm spring months (in Tunisia) [60]
Schistosomiasis	Chronic	b and c	Transmission is seasonal; two seasonal peaks per year (in Tanzania) [61]
Scrub typhus	Acute	a, c, and f	Seasonality depends on activity of vectors (i.e., chiggers) and humans. Seasonality varies geographically. Some areas (in Japan) have strong seasonal transmission, and others have relatively stable transmission [62]
Shigella	Acute	c	Elevated incidence in summer (in Massachusetts, US) [63]
Smallpox	Acute	c	Associated with dry weather [64]
Soil-transmitted helminth infections	Chronic	c and g	Hookworms undergo seasonal arrested development, which affects the acquisition of infection in humans; there is also seasonal acquisition of roundworm infections [65, 66]
Syphilis	Chronic	f	Higher incidence in summer (in China) [67]
Taeniasis (cysticercosis)	Chronic	b and f	Seropositivity varies seasonally in livestock, which are the source of human infection (in Romania) [68]
Tetanus	Acute	c and f	Peak in midsummer (in the US) [69]
Trachoma	Acute and chronic	a	More common in the wet season when the fly vector is most abundant (in Australia) [70]

Since seasonal timing may differ among geographic areas, study location is indicated in parentheses.

*Indicated by author.

**Abbreviations:** MERS-CoV, Middle East respiratory syndrome coronavirus; RSV, Respiratory Syncytial Virus.

**Table 4:** Seasonality of human infectious diseases (continued from Tables 1–3). Drivers categorized as being related to (a) vector seasonality, (c) seasonal climate (e.g., temperature, precipitation, etc.), (h) seasonal flare-up/symptoms and/or remission/latency, (i) observed seasonal incidence with no hypotheses regarding drivers.

Infection/disease	Type	Seasonal driver(s)	Description
TB	Chronic	c and h	Approximately 24% more TB notifications in the summer verses the winter (in the UK) [71]
Typhoid fever	Acute	i and c*	Peaks around July (in China) [72]
Viral meningitis	Acute	i	Higher in the summer, when enterovirus transmission peaks (in Israel) [73]
West Nile virus	Acute	a and c	Peaks July through August in the temperate zones of the Northern Hemisphere [74]
Yaws	Chronic	h	More cases in the wet season; hypothesized to be due to more clinical relapse during the wet season; transmission may be relatively constant throughout the year [75]
Yellow fever	Acute	a and c	Seasonal changes in the distribution and density of the vector *Aedes aegypti*; transmission peak was historically in autumn (in the Americas) [76]
Zika	Acute	a and c	Seasonal changes in incidence are expected to be driven by seasonal fluctuations in the vector population (the *A*. *aegypti* mosquito) [77]

Since seasonal timing may differ among geographic areas, study location is indicated in parentheses.

*Indicated by author.

**Abbreviation:** TB, tuberculosis.

Seasonal variation in infectious disease transmission plays an important role in determining when epidemics happen; however, it is not the sole determinant. For instance, some infectious diseases with known seasonal transmission, such as pertussis and measles, can display multi-annual outbreaks, meaning their epidemics occur in multi-year intervals, such as every two or four years, rather than annually. This is because the timing of these epidemics is determined by a combination of (i) seasonal transmission and (ii) different processes shaping the number of susceptible individuals in the population, a sufficient number of which is a prerequisite for an outbreak.

Within the fields of infectious disease ecology and epidemic modeling, seasonal variation in transmission is known as seasonal forcing [78]. Over the past century, attention has been paid to detailing the cyclicity and mechanisms of seasonal forcing for a few diseases of public interest, such as measles, influenza, and cholera (e.g., see contemporary work by [3, 79, 80]). Despite these notable examples, disease seasonality has yet to be systemically and/or rigorously characterized for the majority of infections.

Here, I aim to motivate future studies of disease seasonality by drawing attention to the importance of seasonality in public health, medicine, and biology. I will explore documented seasonal cycles in human infections, including notifiable and neglected tropical diseases. I also aim to present a holistic view of hypothesized drivers of seasonality for each disease, with the caveat that, for the majority of infections, the current state of the science is insufficient to draw conclusions about seasonal timing, seasonal magnitude, and geographic variation in incidence. Although published data regarding disease seasonality are limited for individual diseases, collectively the body of work on disease seasonality is vast and reveals that infections—which may differ enormously in their pathology and/or ecology—coalesce via underlying seasonal drivers.

In order to explore documented seasonal cycles in human infections, the websites of the United States Centers for Disease Control and Prevention (CDC), World Health Organization (WHO), and the European Centre for Disease Prevention and Control were searched to compile a list of 60+ communicable diseases of public health interest. Careful attention was paid to include neglected tropical diseases that may be underrepresented in disease notification systems. For each infection, online WHO disease information pages were used to determine whether the disease is acute or chronic. When the nature of the infection could not be established from the WHO disease information page, this information was gathered from online CDC disease factsheets. Google scholar was then used to systematically search for information regarding disease seasonality. For each of the diseases, a search was conducted using "[disease name] AND season." When needed, I also added "AND human" to the search term for zoonotic diseases. When searches needed to be more specific, a search for "[disease name] AND seasonality" was conducted. Most diseases had very few papers that specifically focused on seasonality (based on their titles and abstracts); the most relevant paper(s) presented in top search results were used in Tables 1–4. The method employed here was meant to provide a broad overview of many infections as opposed to detailed information regarding any individual infection. A short description of the seasonality and hypothesized seasonal drivers were then summarized in Tables 1–4.

In the broadest sense, seasonal drivers can be separated into four categories: (1) environmental factors, (2) host behavior, (3) host phenology, and (4) exogenous biotic factors. These seasonal drivers may enter into disease transmission dynamics by way of hosts, reservoirs, and/or vectors. In surveying the literature to gauge the breadth of seasonal drivers acting upon human infectious disease systems (Tables 1–4), specific seasonal drivers were found to include (a) vector seasonality, (b) seasonality in nonhuman animal host (i.e., livestock, other domestic animals, or wildlife), (c) seasonal climate (e.g., temperature, precipitation, etc.), (d) seasonal nonclimatic abiotic environment (e.g., water salinity), (e) seasonal co-infection, (f) seasonal exposure and/or behavior and/or contact rate, (g) seasonal biotic environment (e.g., algal density in waterbodies), and (h) seasonal flare-ups/symptoms and/or remission/latency.

### Environmental factors

Environmental factors, specifically climate conditions, are the seasonal drivers that have received the most attention. This may be because they often covary with seasonal disease incidence. Environmental drivers are abiotic conditions that influence transmission via their effects on hosts and/or parasites; classic examples are temperature and rainfall, which influence a variety of infectious diseases [81], but other examples include seasonal nonclimatic abiotic environmental conditions, such as water salinity, which may impact water-borne pathogens. Environmental factors can impact pathogen survival during transitions between hosts. Transitions can take place during short time windows (e.g., for droplet-transmitted infections) or long time windows (e.g., for parasites with environmental life stages). In addition to their impact on pathogens, environmental drivers can also influence host susceptibility to infection or vector population dynamics.

As for host susceptibility, environmental conditions can impact the host immune response and increase cells' susceptibility to infection [82] or pose seasonal challenges (such as food limitations) that leave hosts vulnerable to infection or pathology [83], which has been proposed to influence disease progression in individuals infected with HIV [35]. For directly transmitted infections, environmental conditions can be major drivers of cycles in incidence, with influenza and cholera transmission being notable examples (e.g., see [3, 80]). The effects of climate on flu transmission have been studied using population-level data coupled with transmission models, as well as empirical animal studies [84], to demonstrate the effects of temperature and humidity on transmission. Although climate conditions undoubtedly play a direct role in several directly transmitted infections, they may play a more nuanced role in vector-borne disease systems in which they modulate vector population dynamics and subsequently disease transmission. For example, in the case of African sleeping sickness (Table 1), the rainy season is hypothesized to modify tsetse fly distribution, which results in changes in human–tsetse fly contact and subsequently African sleeping sickness incidence; in this case, we can classify the seasonal driver as (1) vector seasonality alone or as (2) seasonal climate influencing vector seasonality and vector seasonality having a downstream effect on seasonal exposure. Abiotic and biotic seasonal drivers are therefore interconnected and not mutually exclusive.

### Host behavior

Transmission seasonality is sometimes due to seasonal host behavior, specifically fluctuations in transmission-relevant host contact rates throughout the year. Seasonal host behavior not only includes seasonal behavior and/or exposure and/or contact rates in humans but also seasonality in nonhuman animal hosts (i.e., livestock, other domestic animals, or wildlife). The most well-known example of seasonal contact rates in humans is the aggregation of children in schools during school terms that results in elevated transmission of measles (e.g., [1]). Recent studies of seasonal contacts include the use of innovative data sources such as light-at-night satellite imagery and mobile phone data to infer human mobility patterns throughout the year [48, 59]. Data required to quantify seasonal variation in human contact rates are becoming increasingly available. For zoonotic diseases, however, it is contact with wildlife or livestock that can drive seasonal transmission, such as with anthrax, Crimean-Congo hemorrhagic fever, Ebola, echinococcosis, and others (Tables 1–4). Characterizing the seasonal interface among humans and wildlife/livestock poses unique challenges, especially when contacts are occurring in remote areas, which is likely the case with Ebola and echinococcosis (Table 1).

From an ecological perspective, wildlife systems offer a rich arena to study the diverse ways in which behavior impacts disease transmission. Understanding transmission within wildlife is not only relevant for zoonotic infections but is also important for conservation ecology (i.e., in order to protect populations from disease-induced declines, such as is currently experienced by Tasmanian devils, North American bats, and amphibians worldwide). More broadly, understanding the ecology of disease transmission in wildlife can lead to insights that may be applied to human health. Wildlife and urban human populations exist at opposite extremes of a continuum of exposure and/or subjection to natural environmental cycles. Due to the potentially more extreme effect of natural environmental cycles on wildlife disease systems—including climatic influences on wildlife behavior—wildlife could serve as a model for developing methods and conceptual frameworks needed to disentangle the contribution of environmental cycles and behavior from other drivers of disease transmission.

For example, wildlife may be a useful study system for sexually transmitted infections (STIs). Unlike humans, who might have moderate fluctuations in sexual contacts throughout the year [85], in some mammal species, there is a complete absence of sexual contact (and thus transmission) outside of the breeding season. I propose that isolation in time, such as this, is a much more extreme form of seasonal forcing than seen in human infectious disease systems. Isolation in time could also occur for parasites with other transmission modes, in addition to STIs. Although it is an unexplored area of research, the study of isolation in time may reveal pathogen metapopulation structure and parasite adaptations for surviving through transmission troughs. Discrete windows of transmission are likely to have evolutionary consequences for parasite life history. Cattadori and colleagues [86] pointed out that, when transmission is restricted to a short seasonal window, natural selection will favor parasites with "long-lived infective stages." I further speculate that transmission isolated in time will have dynamical consequences that make these disease systems unique from those with more continuous transmission cycles.

Sexual contact is not the only transmission-risk–elevating behavior that can display seasonality. Seasonal engagement in risk-taking behavior may occur in other disease contexts, including for infections transmitted during bouts of fighting. For example, the transmission of facial tumor disease among Tasmanian devils—which is caused by an infectious cancer—is facilitated by aggressive behavior. During a fight, the cancer cells from a facial tumor of an infected devil can be transferred into the wounds and mouth of a susceptible devil, resulting in infection. The Tasmanian devil contact network varies between the mating and nonmating seasons, and this could influence the transmission of this infectious cancer [87, 88]. Although mating and aggression can elevate disease risk, it is important to acknowledge that some behaviors can also mitigate disease risk. In wildlife, disease mitigation behaviors include grooming to remove ectoparasites (as observed in birds and primates) and self-medication (as observed in primates, birds, and monarch butterflies) [89, 90]. There are, however, very few studies of seasonality in risk-taking and risk-mitigation behavior. This is yet another area in which wildlife could serve as a useful model system.

As previously noted, mobility patterns are an aspect of host behavior that can also seasonally structure disease risk via the geographic localization of hosts. In humans, the most notable example is the movement of people in and out of cities. In Niger, for instance, the resulting changes in population density from migration is believed to be the primary driver of measles transmission seasonality [48]. Similarly, in wildlife, hosts seasonally engage with different aspects of their environment. For hosts that migrate or hibernate, contact with risky environments can be seasonal [91]. Migration and hibernation are part of host phenology, which has been implicated in several infectious disease systems [91–94].

### Phenology

Host phenology includes host life history, annual cycles (e.g., migration and hibernation), and endogenous circannual rhythms (i.e., endogenously driven seasonal changes in physiology) [94]. Relevant host phenology includes, but is not limited to, seasonal changes in reproduction, seasonal restructuring of immunity, cycles of metabolism and body condition, hibernation, and migration. Phenology is not only a feature of hosts but also of reservoirs, vectors, and some parasites themselves (particularly helminths). Unlike environmental drivers and host behavior, which can affect diseases dynamics by (i) seasonally forcing transmission in hosts, reservoirs, and vectors, phenology can drive seasonality via additional mechanisms of action, which include the modulation of (ii) susceptible recruitment (via reproduction), (iii) susceptibility to infection, (iv) infectiousness, (v) the recovery rate, (vi) the mortality rate (both natural and disease-induced), and (vii) symptomatology and/or pathology.

Each of the seven mechanisms of action could leave a unique imprint in long-term incidence data, as proposed in [94]. These mechanisms and their drivers, therefore, would have different consequences for disease dynamics. Using models, such as that schematized in Fig 1, statistical inference and simulation studies could be conducted to identify the dynamical effects of various seasonal mechanisms acting in isolation and/or in combination. Simulation studies could provide a foundation for determining the types of data required for distinguishing among seasonal mechanisms and/or drivers and how factors such as resonance can influence the ability to detect seasonal drivers. For example, Martinez-Bakker and colleagues [94] used a simulation study to demonstrate the demographic and transmission regimes under which human birth seasonality is expected to have a meaningful impact on measles incidence in the face of strong seasonal forcing from transmission during school terms.

This challenge of identifying seasonal drivers and their mechanisms of action becomes greater when considering chronic infections, vector-borne diseases, and parasites with complex life histories and phenology of their own. In reviewing seasonal drivers of human disease systems for Tables 1–4, human phenology seemed to be particularly relevant for diseases that have seasonal flare-ups/symptoms and/or remission/latency; this includes some chronic infectious diseases, such as tuberculosis and yaws, along with Meningococcal disease, which is acute (Tables 2–4). Although the study of human phenology is a relatively new research area, phenology of vectors and nonhuman animals is well studied and could be the most common cause of vector seasonality and seasonality in nonhuman animal hosts, which impact diseases such as Zika (Table 4) and Middle East respiratory syndrome coronavirus (MERS-CoV) (Table 3).

### Exogenous biotic factors

In addition to abiotic, behavioral, and phenological features of host–parasite systems, hosts and their parasites are embedded within ecological communities that have additional seasonal aspects. We can refer to biotic factors driven by ecological communities as exogenous biotic factors because they are exogenous to any given host–parasite dyad, host–vector–parasite triad, or multi-host system. Exogenous biotic factors include (1) interactions that take place within hosts—specifically parasite–parasite interactions—and (2) interactions within the ecological community of hosts, reservoirs, and vectors.

During some co-infections, parasite–parasite interactions can occur directly or be mediated via the host immune system, when parasite species impact each other's population dynamics indirectly via their effect on the host immune system. Parasite–parasite interactions can result in parasite fitness being elevated (i.e., facilitation) or dampened (e.g., competition) [95–97]. The seasonality of individual infectious diseases likely imposes seasonal structure on co-infections and thus the presence of parasite–parasite interactions, as has been implicated in the seasonality of bacterial pneumonia (Table 1).

Outside of the host, community ecology becomes particularly relevant for disease transmission in multi-host systems, such as Lyme disease [95, 96, 98]. Community ecology is particularly important when there is heterogeneity in host and/or vector competence in a multi-species disease system. This is because the abundance of competent hosts and/or vectors can determine the transmissibility and maintenance of infection [98]. Each host and/or vector in a vector-borne or multi-host disease system has its own set of ecological interactions (e.g., competitive, commensal, parasitic, etc.) that can indirectly affect the disease of interest. The phenology of hosts, reservoirs, and vectors will give rise to seasonal changes in ecological community composition. Taken together, parasite–parasite interactions within hosts and ecological interactions outside hosts undoubtedly display seasonality, as do nearly all aspects of ecology, and this can influence the dynamics of diseases of public interest.

### Understanding seasonality

Seasonality is an inherent feature of ecological systems, and seasonal incidence is a feature of both acute and chronic infectious diseases (Tables 1–4). It is, therefore, important to conceptualize the epidemic calendar (Fig 1) from the lens of "everything is seasonal." The utility of this lens is that it forces us to carefully consider mechanisms behind disease seasonality, thus preventing what could, in some cases, be the misleading establishment of correlative relationships between seasonal phenomena and infectious disease incidence. Focused attention on building theory that will provide a deeper understanding of seasonal mechanisms and learning how to identify imprints of seasonal drivers in disease data could bring rapid advancement to the field of disease seasonality. In general, if "everything is seasonal," then everything will covary (usually with some phase shifts). Therefore, seasonal covariance alone is not useful for establishing seasonal drivers. Instead, long-term parallel data of potential seasonal drivers and disease incidence should be confronted with mechanistic transmission models. There is evidence to suggest that the information contained in interannual variation and anomalous years holds a key to establishing causal inference of seasonal forcing [99].

A thought experiment can be used to illustrate how anomalous years and interannual variation could be used to establish causal mechanisms of disease seasonality. Let's consider a human disease with peak incidence in the summer, such as polio [6]. Because incidence peaks in summer, it would have a strong positive relationship with temperature, photoperiod (day length), and many other summer-related features of the environment and human populations. To highlight how noncausal seasonal factors could misleadingly correlate with disease incidence, we could quantify what would be a likely strong positive relationship between disease incidence and the sale of summer-related items that have nothing to do with transmission (e.g., bathing suit or ice cream sales). Let's imagine we build a transmission model for this infection and test three potential seasonal drivers (e.g., temperature, photoperiod, and bathing suit sales). We find that all three model variants capture the seasonal structure of the epidemics because they all contain a covariate with the necessary seasonal structure. However, if the disease displays (1) interannual variation in epidemic size and/or (2) anomalous years with differences in epidemic timing that cannot be explained by demography or susceptible recruitment dynamics, then only the model with data from the causal driver would improve our ability to predict the variation in incidence observed among years.

By confronting disease incidence data with transmission models and testing the relevance of various demographic, ecological, behavioral, and physiological covariates, we could identify potential seasonal drivers on the population level. For some seasonal drivers, such as seasonal changes in host immunity, we would, however, still be tasked with understanding the mechanism of action within the host. The effects of seasonal drivers are multi-layered. To better understand seasonality, we must work at multiple organizational levels of science. Geophysical factors, host population ecology, and within-host biology will need to be integrated in the practice of studying seasonality. As previously mentioned, many infectious diseases, which might differ greatly in multiple aspects of their biology, can share the same seasonal driver(s). An immediate way to advance the field of disease seasonality is to leverage the rich weekly and/or monthly datasets available for notifiable diseases and combine them with models and data on potential drivers. By coupling models and data, hypothesis testing can be done to assess seasonal drivers and their modes of action. These data and models can be applied to multiple disease systems in parallel. For instance, parallel study of the seasonal drivers of (a) flu, respiratory syncytial virus (RSV), bacterial pneumonia, and pertussis, or (b) polio, typhoid, and rotavirus, or (c) Zika, dengue, chikungunya, and yellow fever would be a logical start. Uncovering the mechanisms of seasonality for disease systems would empower the public health community to better control infection. This sentiment was shared in 1949 by polio epidemiologist H. Gear, who wrote: "It must be admitted that the reasons for the seasonal incidence of poliomyelitis remain obscure. When they have been elucidated perhaps much of the epidemiology of this disease will be solved" [100].
